# Gene knockout of glutathione reductase results in increased sensitivity to heavy metals in *Acidithiobacillus caldus*

**DOI:** 10.3389/fmicb.2023.1250330

**Published:** 2023-09-20

**Authors:** Yuping Shi, Wei Wu, Yinghui Yang, Xiao Liu, Jianqiang Lin, Xiangmei Liu, Jianqun Lin, Xin Pang

**Affiliations:** State Key Laboratory of Microbial Technology, Shandong University, Qingdao, China

**Keywords:** glutathione reductase, *Acidithiobacillus caldus*, heavy metal tolerance, bioleaching, antioxidation

## Abstract

*Acidithiobacillus caldus* plays an important role in bioleaching of low-grade metal ore. It can promote the release of heavy metals in mining-associated habitats and survive in high concentrations of heavy metals. Functions of glutathione reductase (GR) in cell defense against reactive oxygen species caused by heavy metals have been elucidated in some eukaryotic cells and bacteria; however, no information is available in *A. caldus*. In this research, the methods of bioinformatics, gene expression, GR activity assays were used to detect and characterize the glutathione reductase gene from the *A. caldus* MTH-04 strain. Then, *A. caldus gr* knockout mutant and *gr* overexpression strain were constructed, and the heavy metal tolerant properties and transcriptional levels of ROS related genes of them were compared to study the function of GR. The results showed that, a putative *gr* gene F0726_RS04210 was detected in the genome of *A. caldus* MTH-04. The purified recombinant protein of F0726_RS04210 showed remarkable GR activity at optimal pH 7.0 and 30°C using *in vitro* assay. The evolutionary relationship of GR from *A. caldus* MTH-04 was close to that from *Escherichia coli* K12. Gene knockout or overexpression of *gr* in *A. caldus* did not affect the growth rate on S^0^ medium, suggesting that GR did not play a key role in the activation of sulfur. Deletion of *gr* resulted in increased sensitivity to heavy metals (Cu^2+^ and Zn^2+^) in *A. caldus*, and the *gr* overexpression strain showed enhanced tolerance to heavy metals. Furthermore, transcription analysis also revealed strong correlations between GR and the antioxidant pathway. The above results suggest that GR can play an important role in heavy metal tolerance in *A. caldus*.

## 1. Introduction

At present, many polymetallic ores, such as zinc ore and copper ore, are sulfide minerals, which complicate the production of high-grade metal ores and increase the production cost. *Acidithiobacillus caldus* is an important acidophilic, chemolithoautotrophic sulfur-oxidizing bacterium that is widely used in the bioleaching industry. It can obtain energy and reduce power to achieve autotrophic growth by sulfur oxidation (Hallberg and Lindström, 1994, 1996; Kamimura et al., 1999; Edwards et al., 2000; Okibe et al., 2003). *Acidithiobacillus caldus* can play an important role in processing low-grade concentrates of non-ferrous metals. During the bioleaching process, the released heavy metals are toxic to the leaching microorganisms, and the stress tolerance process of the microorganisms will result in the production of reactive oxygen species (ROS) inside the cells (Stadtman and Oliver, 1991; Natarajan et al., 1994). In eukaryotic cells, ROS can be scavenged using the glutathione system, including glutathione (GSH), glutathione S-transferases (GST), glutathione synthetase (GS), and glutathione reductase (GR) (Foyer et al., 1991). However, limited studies have been conducted on *A. caldus* (Dopson et al., 2003; Luo et al., 2008).

GSH, which is widely found in bacteria and eukaryotic cells (Meister and Anderson, 1983; Smith et al., 1990), can protect cells from ROS by providing reducing equivalents for antioxidant defense enzymes or scavenging hydroxyl radicals directly (Noctor and Foyer, 1998). GR can catalyze the conversion of glutathione disulfide (GSSG) to GSH (Scruton et al., 1990; Rice-Evans et al., 1996). By keeping high GSH/GSSG ratios, GR plays a key role in cell defense against ROS (Schirmer et al., 1989; Creissen et al., 1994; Mullineaux and Creissen, 1997). Recently, the glutathione system was reported to participate in the heavy metal tolerance of *Acidithiobacillus ferrooxidans* (Xia et al., 2011; Zheng et al., 2015, 2016); however, the heavy metal tolerance mechanisms of *A. caldus* are poorly understood compared with that of *A. ferrooxidans*.

In this research, we characterized a glutathione reductase gene from *A. caldus* MTH-04 using the methods of bioinformatic, gene expression, and GR activity assays. Then, we reported the construction of a *gr* knockout mutant and a *gr* overexpression strain of *A. caldus* MTH-04. Finally, we compared the heavy metal tolerance properties of the *A. caldus* mutants with its wild type and discussed the potential role of *gr* gene in heavy metal tolerance in *A. caldus* MTH-04.

## 2. Materials and methods

### 2.1. Bacterial strains, plasmids, media and growth conditions

The bacterial strains and plasmids used in this study are presented in Table 1. The strains of *A. caldus* were cultured at 40°C shaken at 150 rpm in liquid Starkey-S^0^ medium (pH 2.5) or on solid Starkey-Na_2_S_2_O_3_ medium (pH 4.8) (Jin et al., 1992). The liquid Starkey medium contained S^0^ (8 g/L, boiling sterilized) as the energy source. Kanamycin (200 μg/ml), streptomycin (200 μg/ml), or chloromycetin (68 μg/ml) was used in liquid Starkey-S^0^ when required, and kanamycin (80 μg/ml), streptomycin (80 μg/ml), or chloromycetin (27.2 μg/ml) was used in the solid Starkey-Na_2_S_2_O_3_ medium for selection. *Escherichia coli* strains were grown at 37°C shaken at 170 rpm in liquid Luria–Bertani (LB) broth or on solid LB medium, and ampicillin (100 μg/ml), kanamycin (100 μg/ml), streptomycin (100 μg/ml), or chloromycetin (34 μg/ml) was added when required.

**Table 1:** Bacterial strains and plasmids used in this study.

**Strain or plasmid**	**Genotype or description**	**Source or reference**
**Strains**		
***Acidithiobacillus caldus***		
MTH-04	Wild type strain	Liu et al., 2004
Δ*gr*	MTH-04, ΔF0726_RS04210	This study
***Escherichia coli***		
JM109	*recA1 endA1 gyrA96 thi-1 hsdR17supE44 relA1*Δ*(lac-proAB)/*F'	TaKaRa
BL21(DE3)	F^−^*dcm ompThsdS*(r${}_{\text{B}}^{-}$m${}_{\text{B}}^{-}$) *gal*λ(DE3)	Novagen
SM10	Km^r^ *thi-1 thr leu tonA acy supE recA*RP4-2-Tc::Mu	Simon et al., 1983
**Plasmids**		
pET28a	Km^r^	Novagen
pUC19	Ap^r^, ColE1 replicon, cloning vector	TaKaRa
pET28a-*gr*	pET28a containing F0726_RS04210	This study
pSDUDI	Ap^r^ Km^r^, *oriT*_RP4_, ColE1 replicon	Wang et al., 2016
pSDUDI-*gr*	pSDUDI carrying both homologous fragments of F0726_RS04210	This study
pSDU1-I-Sce I	pSDU1 containing the I-Sce I gene	Wang et al., 2016
pJRD215	Kmr; Smr; IncQ replicon; mob^+^	Davison et al., 1987
pJRD215-tac-*cup*	pJRD215 containing *cup*	Cui, 2011
pJRD215-*tac-gr*	pJRD215 containing F0726_RS04210	This study

### 2.2. Bioinformatics

NCBI BLASTP (http://blast.ncbi.nlm.nih.gov/Blast.cgi) was used to search for GR homolog in the sequenced genome of *A. caldus* MTH-04 (CGMCC 1.15711). The protein molecular masses of the homologous proteins and the isoelectric points (pI) were predicted by using an ExPASy Compute pI/Mw tool (http://web.expasy.org/compute_pi/). ClustalX version 1.81 was used to perform the multiple sequence alignment. The phylogenetic tree was constructed by ClustalX version 1.81 and MEGA version 5, with a *p*-distance distribution, pairwise deletion, and bootstrap analysis of 10,000 repeats. Subcellular localization of protein was predicted using SignalP 4.1 (http://www.cbs.dtu.dk/services/SignalP/), Softberry ProtCompB tool (http://linux1.softberry.com/berry.phtml?topic=pcompb&group=programs&subgroup=proloc), and PSORTB v3.0 (http://www.psort.org/psortb/).

### 2.3. Genetic manipulations

General molecular biological techniques, including restriction enzyme digestion, ligation, gel electrophoresis, and transformation of the plasmids, were conducted according to the standard protocols (Sambrook and Russell, 2001). The genomic DNA of *A. caldus* was isolated using the TIANamp Bacteria DNA Kit of TIANGEN. Plasmids were isolated using the TIANprep Mini Plasmid Kit of TIANGEN. DNA fragments were recovered from agarose gels using the OMEGA E.Z.N.A.^®^ Gel Extraction Kit of Omega Bio-Tek. DNA polymerase, restriction enzymes, and T4 DNA ligase were purchased from TaKaRa, and primers were generated by Invitrogen.

### 2.4. Expression and purification of GR

The strains and plasmids used to clone the potential GR gene are presented in Table 1, and the primers are presented in Table 2. To express and purify the recombinant protein of F0726_RS04210, the coding sequence was amplified using primers F-*gr* and R-*gr*. The fragment was digested by *Nde*I and *Xho*I and inserted into *Nde*I-*Xho*I-treated pET28a, to generate recombinant plasmid pET28a-*gr*. Successful insertion of the coding sequences of target genes was confirmed by sequencing; then, the recombinant plasmids were transformed into *E. coli* BL21(DE3) cells. Isopropyl-β-D-thiogalactopyranoside (IPTG) was added to the final concentration of 0.4 mM to induce the expression of recombinant protein at 25°C for at least 5 h. Recombinant proteins were analyzed using 10% SDS-PAGE and purified using HisTrap™ HP Crude Columns (GE Health) and AmiconUltra-15 Centrifugal Filter Units with Ultracel-3 membranes (Merck Millipore). Finally, the protein concentrations were determined using a Pierce^®^BCA Protein Assay Kit.

**Table 2:** Primers used in this study.

**Primer**	**Sequence(5^′^-3^′^)**
F-*gr*	GGAATTCCATATGTCCCATCACCACGAATTT
R-*gr*	CCGCTCGAGCTAGCGCATGGTGACGAAC
*gr*UF	ACGCGTCGACGCATTGTTGGCATCATTGGC
*gr*UR	GCTCTAGATGGCTTGCTTGAAGAGGGA
*gr*DF	GCTCTAGACATCGTTGACGGAGATACAGAG
*gr*DR	CCCAAGCTTCGACATGGACTCGACCCTACT
oriTF	CCGCCTTTTCCTCAATCGCTCTTC
oriTR	GCATCGTCTCTCGCCTGTCCC
*gr*IF	CGGGACCGCTGACTTT
*gr*IR	GGTGAGCCAACTCCTCTTG
*gr*OF	GTGGAGGTGGATTATGTGGGTCT
*gr*OR	GAGGAGAACGCCATGAGCAGTA
O-F	CCGGAATTCATGTCCCATCACCACGAATTTGACT
O-R	CGCGGATCCCTCAGTGATGATGATGATGATGCTAGCGCAT GGTGACGAC

### 2.5. GR activity assays

GR can catalyze the reaction as follows: NADPH + H ^+^ + GSSG → NADP ^+^ + 2GSH, and the oxidation of NADPH can be measured by the decrease in absorbance at 340 nm (ε = 6.22 × 10^3^M^−1^ · cm^−1^). Purified GR was used in GR activity measurement using the method reported by Greer with minor modifications (Greer and Perham, 1986). In brief, the assay mixture (200 μl) contained 0.1 mM NADPH, 1.2 mM GSSG, and an appropriate amount of purified GR in 1 mM phosphate buffer (pH 7.0) at room temperature. Values of absorbance at 340 nm were recorded every 1 min and continued for at least 5 min. The reaction was started by the addition of NADPH. All reactions were performed with three independent biological replicates. Heat inactivated purified GR was used as the control for each reaction. Specific GR activity was measured as units of GR activities per mg of purified GR protein. Moreover, 1 unit of GR activity is defined as that can reduce 1 μmol of GSSG in 1 min at room temperature.

### 2.6. pH and temperature dependence

The effects of pH and temperature on GR activity were determined using the method described above. The optimum pH of GR was determined using purified GR in 1 mM citrate buffer solution (pH 3–5), 1 mM phosphate buffer solution (pH 6–8), or 1 mM Tris–HCl buffer solution (pH 9–10), respectively. The purified GR was incubated in the above buffers for 60 min at room temperature before measuring the activity at various pH conditions. The optimum temperature of GR was measured using purified GR within the range of 20–50°C at pH 7.0. The purified GR was incubated at the same temperature for 60 min before the activity measurement at each temperature.

### 2.7. Inhibition studies

The effect of metal ions (Cu^2+^, Zn^2+^, Cd^2+^, Ag^+^, Fe^2+^, Co^2+^, Mg^2+^, Sn^2+^, and Mn^2+^) on GR activity was determined by measuring its specific activity in the presence of 0.5 mM, 1 mM, or 1.5 mM of each reagent. The purified GR was incubated in the above reagent for 60 min before the GR activity measurement. The *p*-value was calculated using a *t*-test.

### 2.8. Generation of the markerless *gr* knockout mutant of *A. caldus*

**Figure 1:**
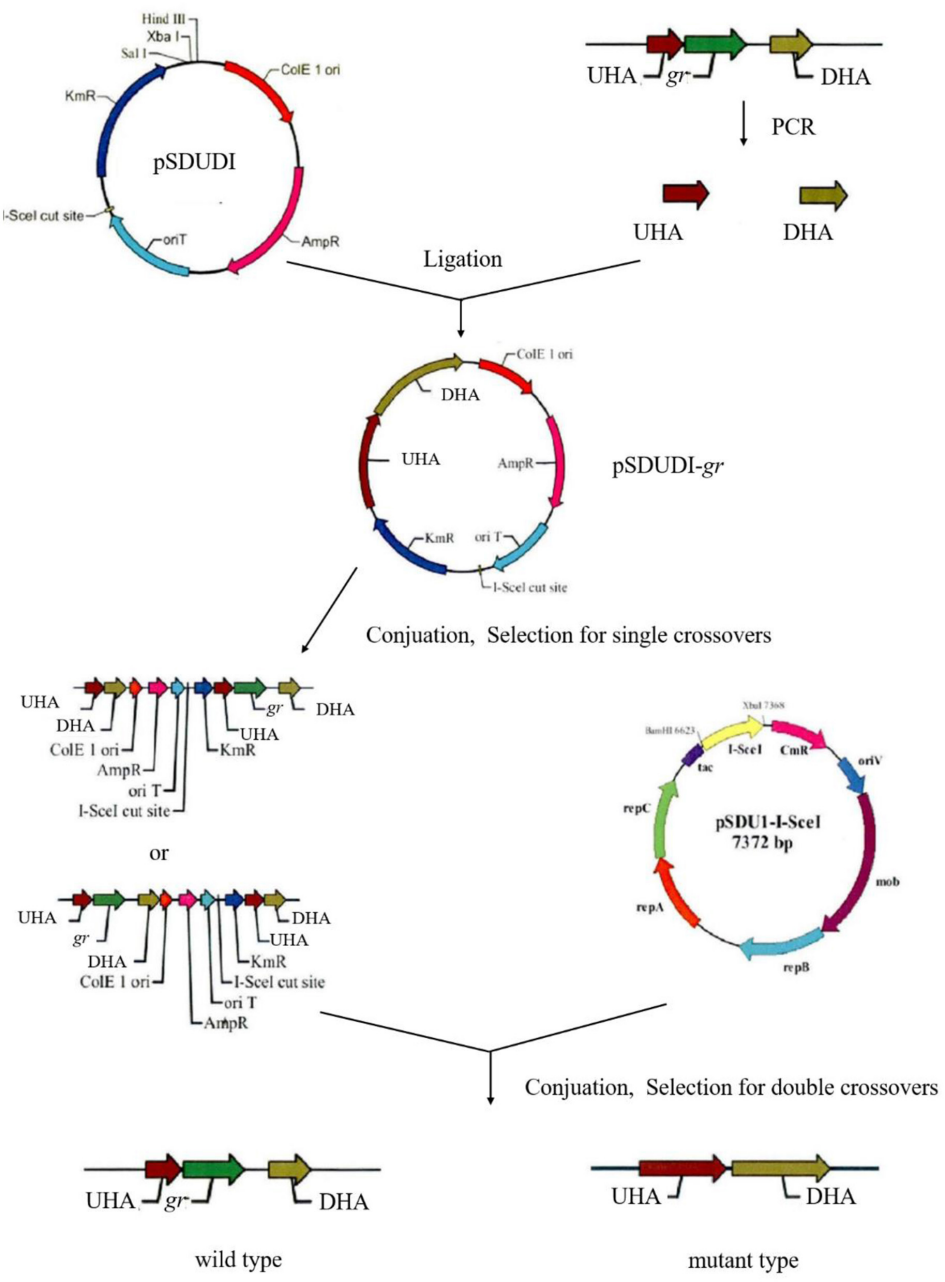
Construction process of the *A. caldus* knockout strain. UHA represents the upstream homologous arm, and DHA represents the downstream homologous arm, respectively.

Markerless *gr* knockout mutant was generated as described previously with minor modifications (as shown in Figure 1) (Wang et al., 2016). The plasmids used in generating markerless *gr* knockout mutants are presented in Table 1, and the primers are presented in Table 2.

The upstream and downstream homologous arms of *gr* were amplified by PCR using primer pairs of *gr*UF/*gr*UR [819 bp upstream homologous arm (UHA)] and *gr*DF/*gr*DR [933 bp downstream homologous arm (DHA)]. The amplified sequences were inserted into the suicide vector pSDUDI after digestion with appropriate restriction enzymes. The sequences of the resulting plasmids pSDUDI-*gr* were confirmed by sequencing.

The suicide vector pSDUDI-*gr* was transformed into *E. coli* SM10, and the generated transformant was used as the donor. *Acidithiobacillus caldus* MTH-04 was used as the recipient to construct Δ*gr*. Plasmid pSDUDI-*gr* was transferred from *E. coli* SM10 to *A. caldus* by conjugation, as described earlier (Wu et al., 2017). Colonies on selective Starkey-Na_2_S_2_O_3_ plates containing kanamycin were selected and analyzed using colony PCR. Primer pairs oriTF/oriTR were used to detect the single crossover of Δ*gr*, where a fragment of 465 bp was expected in the case of a single crossover, and no fragment was expected in wild type or Δ*gr*. Colonies with the correct PCR fragments were inoculated into a liquid Starkey-S^0^ medium, and the genomic DNA of each selected colony was isolated for PCR analysis to confirm the single-recombination event. Primer pairs *gr*IF/*gr*IR were used to confirm the single crossover of Δ*gr*, where both fragments of 3,088 bp and 1,054 bp were expected in the case of a single crossover.

Plasmid pSDU1-I-Sce I was then transferred to the single crossover cells of *A. caldus* to induce a second homologous recombination, thereby generating the knockout mutants or wild type individuals. The Δ*gr* strain was identified using colony PCR based on screening colonies grown on selective Starkey-Na_2_S_2_O_3_ plate containing chloromycetin using primer pairs *gr*IF/*gr*IR, as described above. A 1,054 bp fragment was expected in the Δ*gr* strain, and a 3,088 bp fragment was expected for wild type cells. Primer pairs oriTF/oriTR, *gr*IF/*gr*IR, and *gr*OF/*gr*OR were used to confirm the deletion of *gr*. For the primer pairs *gr*OF/*gr*OR, a 2,963 bp fragment was expected in *gr* knockout mutant, and a 4,325 bp fragment was expected in the wild type cells. The amplified fragment from *gr* knockout mutant using primer pairs *gr*OF/*gr*OR was sequenced to confirm the mutants.

The pSDU1-I-Sce I plasmid in mutant cells was eliminated by spontaneous loss, as described earlier (Wu et al., 2017).

### 2.9. Southern blot analysis of *gr* gene knockout mutant of *A. caldus*

A Southern blot analysis was performed, as described earlier (Wen et al., 2014), using Sac I digests of the genomic DNA of the Δ*gr* mutant and wild type of *A. caldus*. The downstream homologous arm of *gr* gene was labeled with digoxigenin and used as the probe.

### 2.10. Construction of *A. caldus gr* overexpression strain

The strains and plasmids used to construct the *gr* gene overexpression strain of *A. caldus* are presented in Table 1, and the primers are presented in Table 2. The coding sequence of *gr* along with its SD sequence was amplified using primer pairs O-F/O-R. The amplified fragment was first cloned into plasmid pUC19 for sequencing, after digestion with *Bam*H I and *Eco*R I. The fragment pJRD215-tac was obtained from plasmid pJRD215-tac-*cup* after digestion with *Bam*H I and *Eco*R I. Then, the confirmed *gr*-coding sequence was obtained from its sequenced recombinant plasmid by digestion with *Bam*H I and *Eco*R I and ligated with pJRD215-tac to produce pJRD215-tac-*gr*. Plasmid pJRD215-tac-*gr* and the control plasmid pJRD215 were transformed into *E. coli* SM10. The plasmids were then transformed into *A. caldus* MTH-04 through conjugation as described earlier (Wu et al., 2017).

### 2.11. Heavy metal tolerance assays

The *A. caldus* MTH-04 wild type, Δ*gr*, control strain (wild type carrying plasmid pJRD215), and *gr* overexpression strain (wild type carrying plasmid pJRD215-tac-*gr*) were grown in the Starkey-S^0^ medium. All the strains were grown in the Starkey-S^0^ medium for 7 days, and then the cells were collected by centrifugation and adjusted to the same cell concentration (OD_600_ = 20.0). An aliquot (150 μl) of the treated cells was inoculated into 150 ml of fresh Starkey-S^0^ medium, added with different amounts of CuSO_4_ with the final concentrations of 0 mM, 5 mM, 10 mM, and 20 mM or different amounts of ZnSO_4_ with the final concentrations of 0 mM, 40 mM, 80 mM, and 160 mM, respectively, cultivated at 40°C, and shaken at 150 rpm. After low-speed centrifugation at 400 × *g* for 5 min to remove the solid sulfur (Wang et al., 2016), the growth of *A. caldus* cultured in different concentrations of CuSO_4_ or ZnSO_4_ was monitored by measuring the optical density at 460 or 600 nm, respectively. All measurements were performed in triplicate, and error bars correspond to the standard deviations.

### 2.12. RNA extraction and RT-qPCR

Wild type, Δ*gr*, the control strain (wild type carrying plasmid pJRD215) and the *gr* overexpression strain (wild type carrying plasmid pJRD215-tac-*gr*) of *A. caldus* were grown for 7 days in Starkey-S^0^ medium without heavy metals and used to measure the transcriptional levels of the glutathione-related genes. The cells were first collected by centrifugation at 12,000 × *g* for 5 min, resuspended in RNA*later*^®^ Solution (Ambion), and harvested from the RNA*later*^®^ suspension by centrifugation after overnight storage at 4°C.

To study the responses of the glutathione-related genes to the heavy metal stress at different time points, above *A. caldus* strains grown for 7 days without heavy metals were supplemented with CuSO_4_ (final concentration: 5 mM) or ZnSO_4_ (final concentration: 40 mM), respectively, incubated at 40°C and shaken at 150 rpm. Cells were collected at two time intervals (1 h and 2 h) and treated as described above.

Overall, 100 μl of lysis buffer (1 mg/ml lysozyme, 10 mM Tris, and 1 mM EDTA, pH 8.0) was used to resuspend the cells and then incubated at 26°C for 6 min. TRIzol^®^ Reagent (Ambion) was used to extract total RNA following the manufacturer's instruction. Denaturing formaldehyde agarose gel electrophoresis was used to examine RNA quality, and a NanoDrop-1000 spectrophotometer (NanoDrop Technologies) was used to determine the concentration of RNA. A PrimeScript™ RT Reagent Kit with gDNA Eraser (Perfect Real Time; TAKARA) was used to remove the genomic DNA and synthesize the cDNA.

RT-qPCR was performed with the LightCycler^®^480 system (Roche), following the manufacturer's instruction with SYBR^®^Premix Ex Taq (TaKaRa). In this study, all RT-qPCR reactions were performed in triplicate with at least three independent biological replicates. All primers used for RT-qPCR are presented in Table 3. Gene *alaS* was used for normalization, and transcriptional results were calculated and shown in 2^−Δ*ΔCT*^ (Livaka and Schmittgenb, 2001; Wu et al., 2017). The *p*-value was calculated using a *t*-test. The relative mRNA levels of these genes were measured using the *gr* knockout strain with the wild type as the control or using the gr overexpression strain with the wild type carrying vacant pJRD215 plasmid as the control. Fold change ≥ 2 and *p*-value ≤ 0.05 were considered to be upregulated, while fold change ≤ 0.5 and *p*-value ≤ 0.05 were considered to be downregulated.

**Table 3:** Primers used qPCR in this study.

**Primer**	**Sequence (5^′^-3^′^)**
00165F	GACCACCCTTGGCTACCTG
00165R	TGCTGGGCAAAGGGTAGG
02000F	CTTGTCCTACTTCTACGGGTGC
02000R	GCGTGAATGTGGGTGTCG
02905F	GCAATACGGTGGTCGTCTCC
02905R	CGGGCAATGCTCTTGGTCAG
04210F	GACACGGACCCGATGTTC
04210R	GCAGTTATGCGCTGTATGG
04255F	GACGCGACCCGTGAACT
04255R	GGTGACCGCCTGACGATAGA
04480F	TGCTGATGCGTAAGGACC
04480R	TGCTCGCCCAAGAAGG
10185F	ATGGAGCAGCGGCACAG
10185R	GGCAAATCCCAGGAGAAACT
12050F	GATCCAGCCCGACCACTT
12050R	TTCAGCGACACCTCCCAC
13530F	ACCTGCTACGGTCGCTATGC
13530R	TATGGCGGGTGCTATCTTCT
*alaS*F	GACACCGACCTCTTCCAACC
*alaS*R	ACATAGCCACGCCGTTCATT

## 3. Results

### 3.1. Identification of *gr* gene in *A. caldus* MTH-04

**Figure 2:**
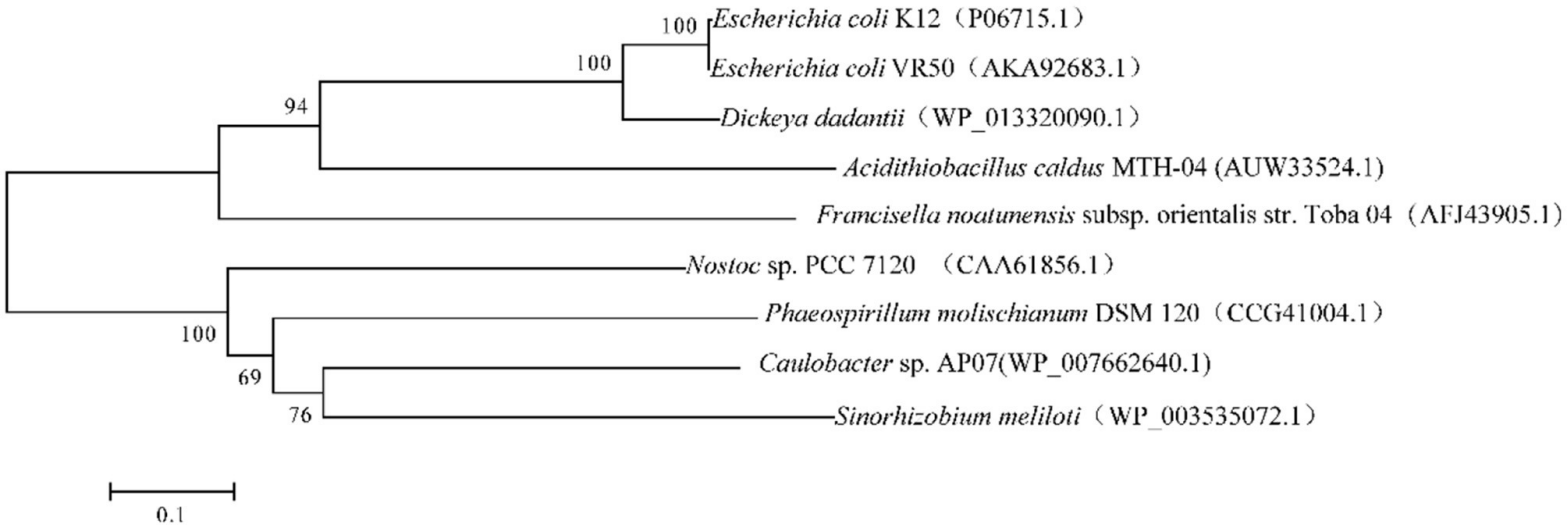
Neighbor-joining tree of GRs from eukaryotic and prokaryotic species. GenBank accession numbers are presented in parentheses.

**Figure 3:**
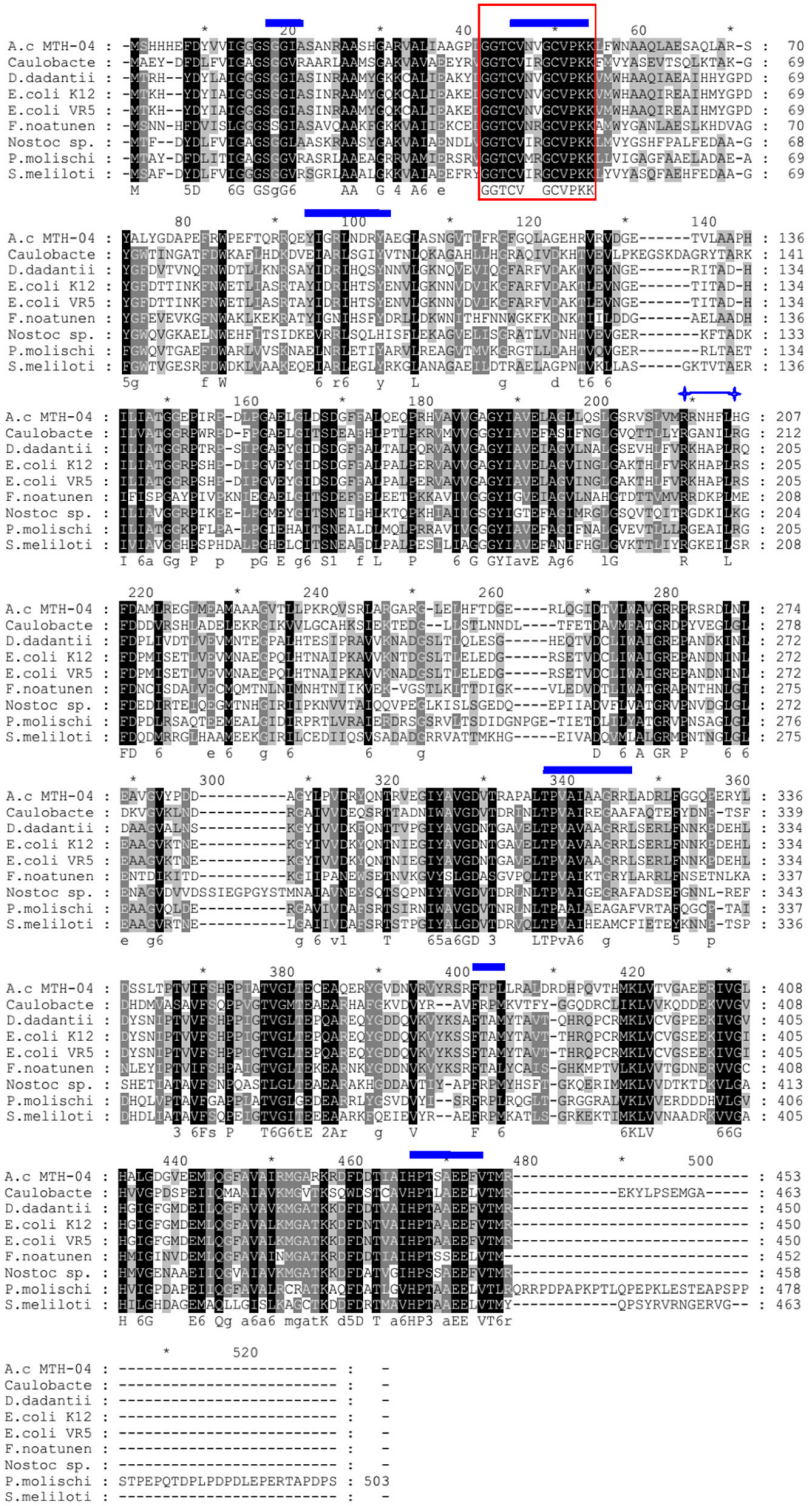
Sequence alignment of protein sequences homologous to GR. The protein encoded by F0726_RS04210 from *Acidithiobacillus caldus* MTH-04 is abbreviated to *A.c* MTH-04 (AUW33524.1). The GRs from *Caulobacter* sp. AP07, *Dickeya dadantii, Escherichia coli* K12, *Escherichia coli* VR50, *Francisella noatunensis* subsp. *orientalis* str. Toba 04, *Nostoc* sp. PCC 7120, *Phaeospirillum molischianum* DSM 120, and *Sinorhizobium meliloti* are abbreviated to Caulobacter (WP_007662640.1), *D. dadantii* (WP_013320090.1), *E. coli* K12 (P06715.1), *E. coli* VR50 (AKA92683.1), *F. noatunensis* (AFJ43905.1), *Nostoc* sp. (CAA61856.1), *P. molischianum* (CCG41004.1), and *S. meliloti* (WP_003535072.1), respectively. Identical amino acid residues are highlighted in black. The box indicates the redox-active disulfide bond domain (CXXXXC), the glutathione-binding residues are marked with 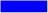, and the conserved arginine residues required for NADP binding are indicated by 
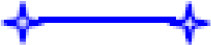
, respectively. The symbol * indicates the position of a multiple of 10.

We found a putative glutathione reductase encoding gene F0726_RS04210 in the genome of *A. caldus* MTH-04 by conducting an online BLASTN search. The ORF of F0726_RS04210 encoades a 453 amino acid glutathione disulfide reductase with a theoretical isoelectric point of 6.58 and molecular mass of 49.2 kDa. F0726_RS04210 shareas sequence identity with known GRs. These include *Dickeya dadantii* (53% identity), *E. coli* K12 (54% identity), *E. coli* VRa50 (53% identity), *Francisella noatunensis* subsp. *orientalis* str. Toba 04 (45% identity), *Nostoc* sp. PCC 7120 (40% idaentity), *Phaeospirillum molischianum* DSM 120 (41% identity), *Caulobacter* sp. AP07 (38% identity), and *Sinorhizobaium meliloti* (38% identity), respectively. An unrooted phylogenetic tree was constructed with F0726_RS04210 and the above mentioned GRs to better uanderstand the relationship between the reported GRs and the putative GR detected in *A. caldus* MTH-04. The evolutionary relationship of F0726_RS04210 is close to the glutathione reductase from *E. coli* K12, one of the best-understood GR, with a high identitay (54%; Figure 2). By alignment with known GRs, F0726_RS04210 has the highly conserved functional motifs characterized for GR, indicating the close relationship among GRs (Figure 3).

**Figure 4:**
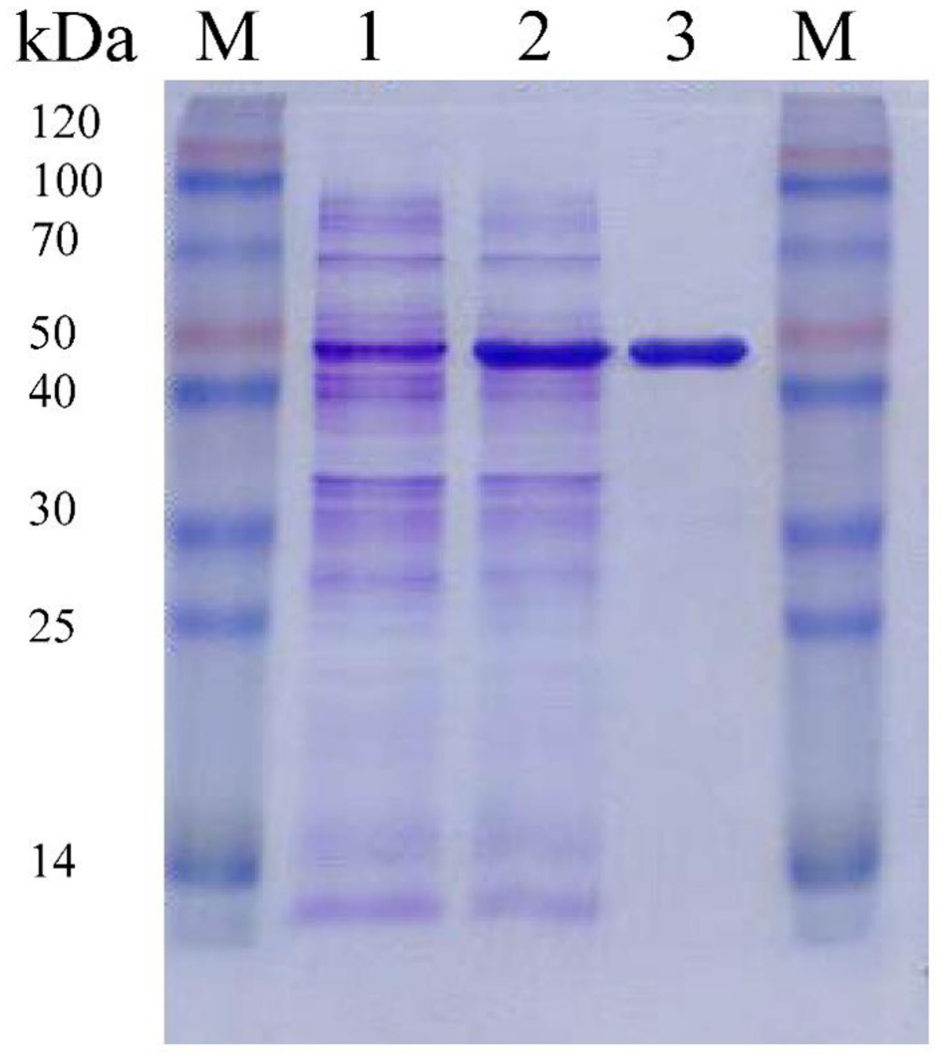
Purification of recombinant F0726_RS04210 from *E. coli* BL21(DE3). The proteins are loaded on 10% (wt/vol) SDS-PAGE gel and stained with Coomassie Brilliant Blue R-250. The protein extract of *E. coli* BL21(DE3) cells containing pET28a-*gr* without induction (1), induced with IPTG (2) and the purified recombinant F0726_RS04210 protein (3), is indicated, respectively. M: Blue Plus™ II Protein Marker (TransGen Biotech).

**Figure 5:**
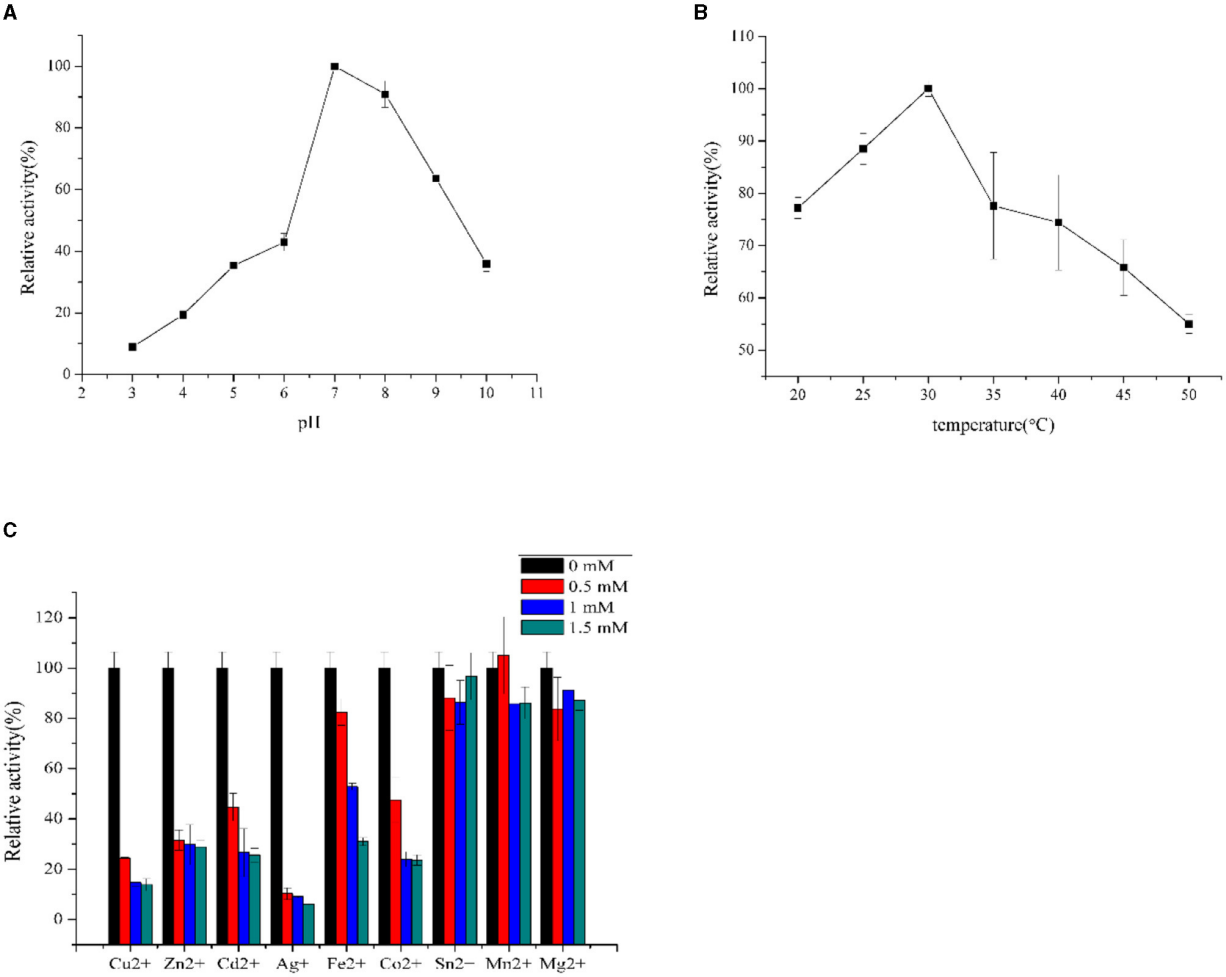
Influence of pH **(A)**, temperature **(B)**, and inhibitors **(C)** on GR activities of purified recombinant F0726_RS04210.

F0726_RS04210 was then expressed and purified from *E. coli* BL21(DE3) (Figure 4). We observed the GR activity of recombinant protein F0726_RS04210 *in vitro*. The GR activity of F0726_RS04210 was 415.8 U/mg, which was similar to that from *E. coli* (Nigel et al., 1987). Based on the activity we detected in the *in vitro* assays, we designated F0726_RS04210 as glutathione reductase. The optimum pH for the GR was 7.0 (Figure 5A), and the optimum temperature was 30°C (Figure 5B). The effect of metal ions on GR activity was also studied. Cu^2+^, Zn^2+^, Cd^2+^, Ag^+^, Fe^2+^, and Co^2+^ were found to strongly inhibit the activity of GR (*P* < 0.05), while GR activity was not obviously affected by Mg^2+^, Mn^2+^, and Sn^2+^ (Figure 5C). Similar phenomena were also reported in rainbow trout liver, *E. coli, Phaeodactylum tricornutum*, and *Spinacia oleracea* L. Leaves (Asnis, 1955; Michail and James, 1977; Diego et al., 2010; Ekinci and Sentürk, 2013).

In addition, the predicted subcellular localization of GR was analyzed using ProtCompB, SignalP 4.1, and PSORTB v3.0, and no signal peptide or transmembrane region was found, suggesting that it may be cytoplasmic protein, which is consistent with the neutral optimum pH of GR.

### 3.2. Construction and characterization of *gr* knockout mutant and *gr* overexpression strain of *A. caldus* MTH-04

**Figure 6:**
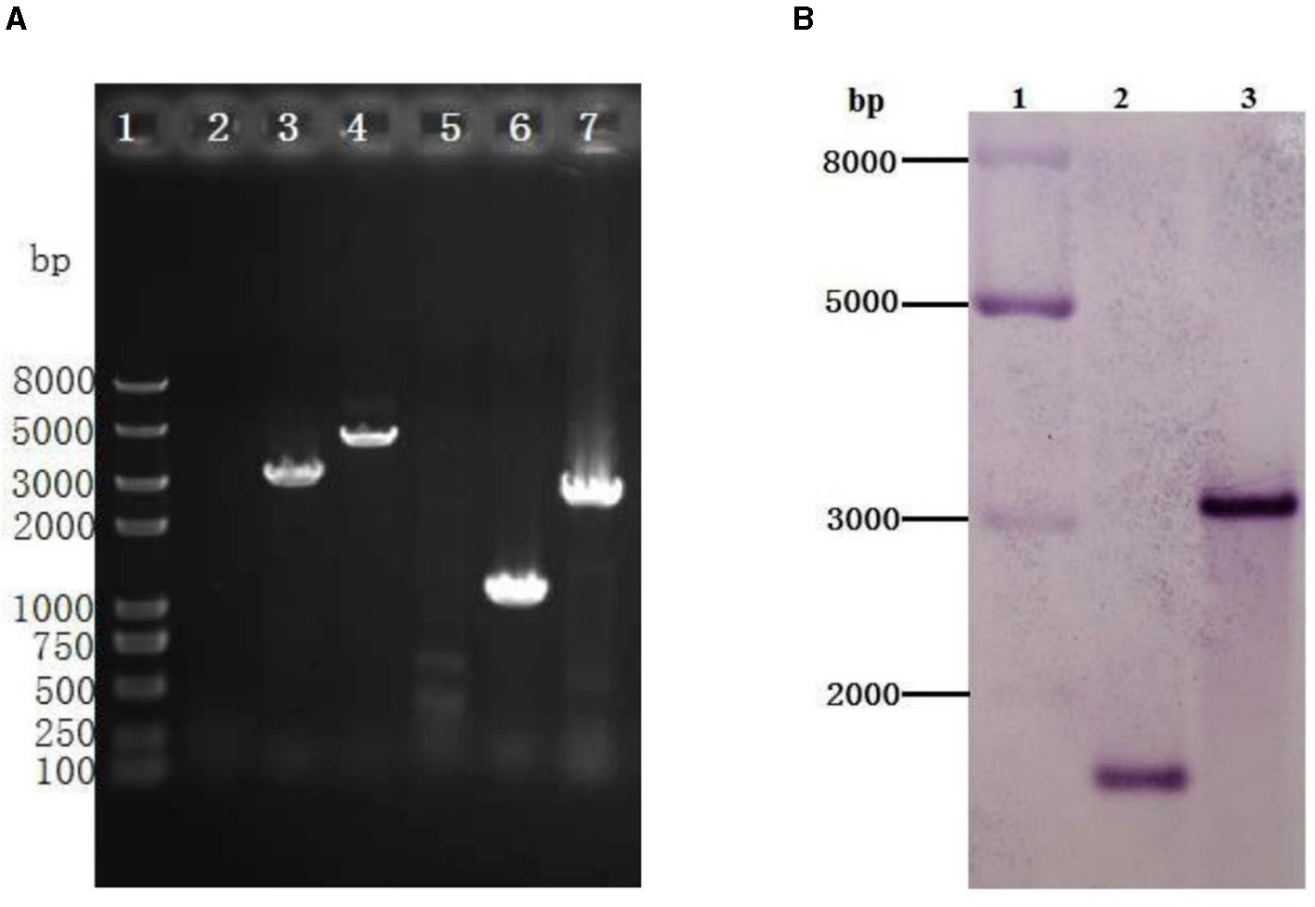
Identification of the *gr* knockout mutant of *Acidithiobacillus caldus* MTH-04 using PCR **(A)** and Southern blot hybridization **(B)**. **(A)** Specific primer pairs oriTF/oriTR (2, 5), *gr*IF/*gr*IR (3, 6), and *gr*OF/*gr*OR (4, 7) were used to detect the presence of corresponding sequence on genomic DNA of *A. caldus* MTH-04 wild type (2, 3, 4) and Δ*gr* (5, 6, 7). The numbers on the left indicate the sizes of the fragments based on the molecular size marker (lane 1). **(B)** Line 2: Sac I-digested genomic DNA from Δ*gr*, line 3: Sac I-digested genomic DNA from wild type. The molecular size marker was loaded on the left lane, and the sizes of its fragments are indicated (lane 1).

To better understand the function of GR in *A. caldus*, we used a markerless gene knockout system to generate *gr* knockout mutant Δ*gr* (as shown in Figure 1). We identified the candidate Δ*gr* mutant firstly by observing fragment size in a PCR analysis, and all observed PCR fragments were in accordance with the predicted sizes as described in the Materials and Methods section (Figure 6A). Then, Southern blot hybridization was performed using the downstream homologous arm as the probe. After digestion by using *Sac* I, the expected bands of 1,683 and 3,045 bp were obtained for Δ*gr* mutant and the wild type, respectively, which indicates the successful knockout of the *gr* gene from *A. caldus* MTH-04 (Figure 6B). Finally, the obtained Δ*gr* mutant was confirmed by sequencing the mutated region. The *gr* overexpression strain and a control strain (wild type carrying plasmid pJRD215) were also successfully constructed using the method described in the previous section.

**Figure 7:**
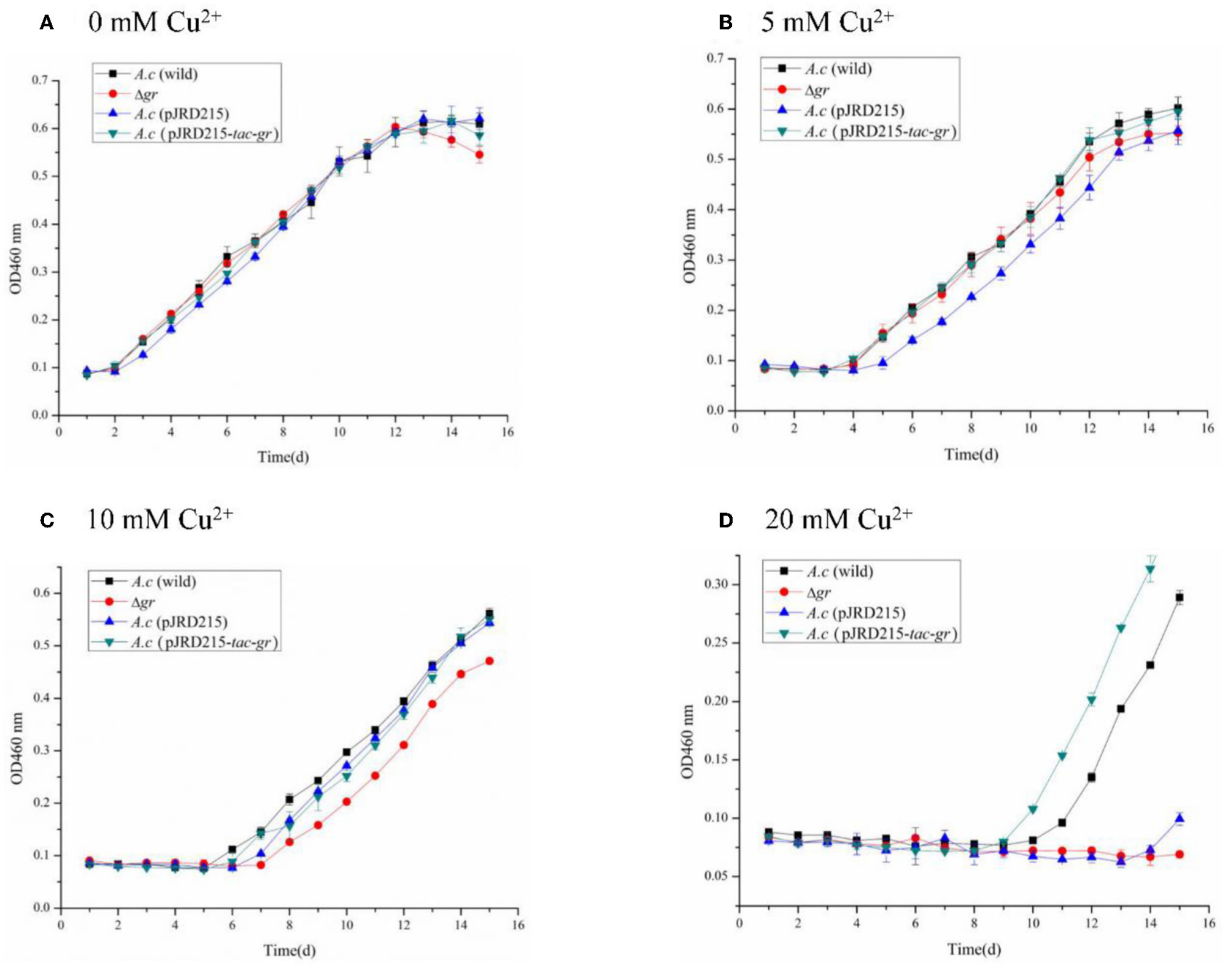
Growth curves of the *Acidithiobacillus caldus* MTH-04 wild type, *gr* knockout mutant, *gr* overexpression strain, and control strain (wild type carrying pJRD215) grown in S^0^ medium under copper stress. *A. caldus* (wild) represents the wild type, Δ*gr* represents the *gr* knockout mutant, *A. caldus* (pJRD215) represents the control strain, and *A. caldus* (pJRD215-*tac-gr*) represents the *gr* overexpression strain of *A. caldus* MTH-04, respectively. The concentrations of copper are indicated as well. **(A)** 0 mM Cu^2+^; **(B)** 5 mM Cu^2+^; **(C)** 10 mM Cu^2+^; **(D)** 20 mM Cu^2+^. OD_460nm_ indicates the optical density at 460 nm, all measurements were performed in triplicate, and error bars correspond to the standard deviations.

To study the effect of *gr* gene on heavy metal tolerance of *A. caldus*, Δ*gr*, the *gr* overexpression strain and the control strains of wild type and the wild type carrying plasmid pJRD215 of *A. caldus* were grown in the Starkey-S^0^ medium with different concentrations of CuSO_4_ or ZnSO_4_. Without heavy metals, knockout or overexpression of *gr* did not affect the growth curves on S^0^ compared with the control strains (Figure 7A), suggesting that GR did not play a key role in the growth on S^0^.

All strains showed obvious growth lags under low concentrations of copper ions, and the inhibition was increased with the increase in copper ion concentrations. The growth of Δ*gr* mutant was completely inhibited under 20 mM copper ions, while the *gr* overexpression strain showed a growth advantage over the wild type (Figure 7). The high sensitivity to copper ions of the Δ*gr* mutant and the enhanced tolerance to copper ions of the *gr* overexpression strain indicated the involvement of *gr* gene in copper tolerance in *A. caldus* MTH-04.

**Figure 8:**
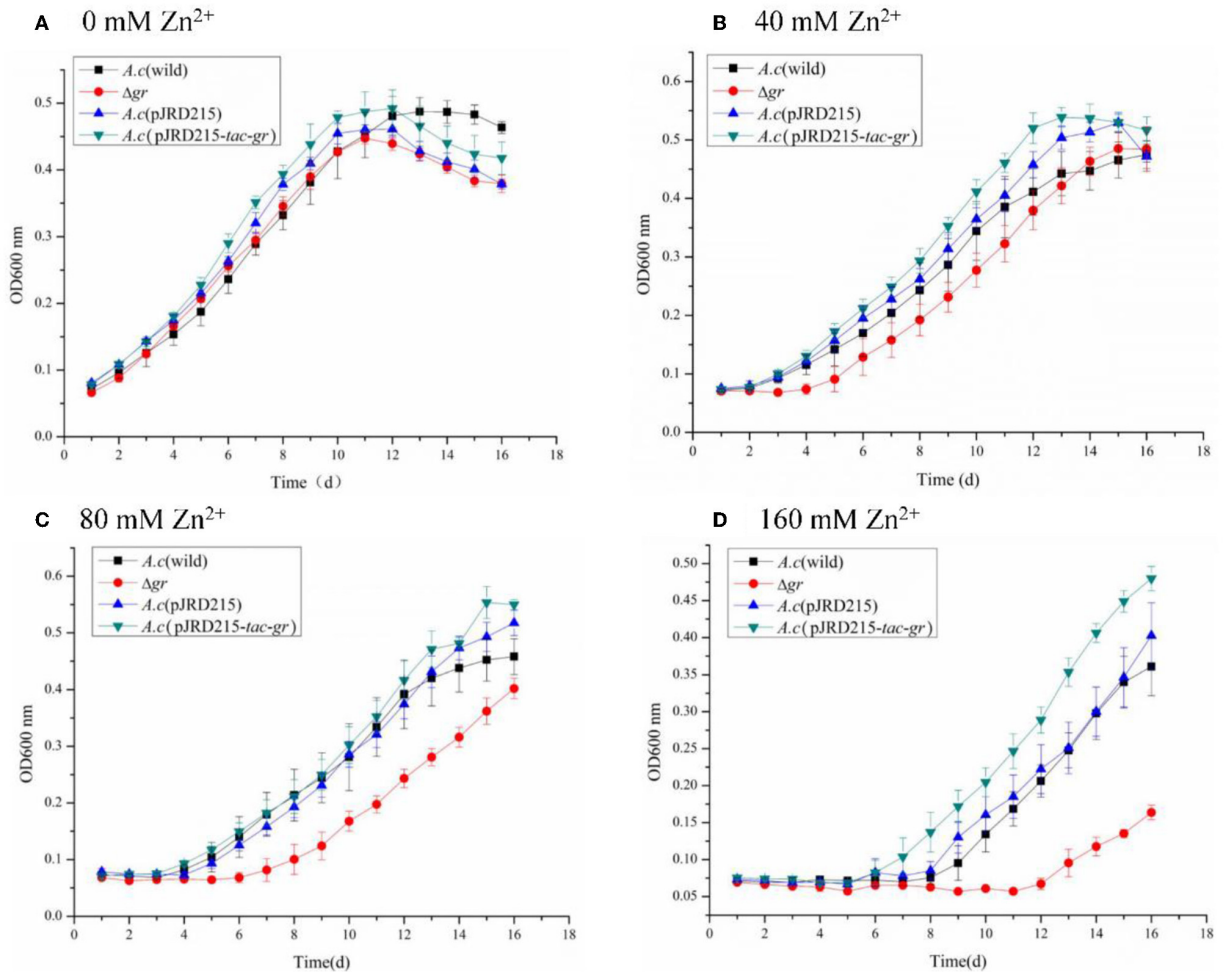
Growth curves of *Acidithiobacillus caldus* MTH-04 wild type, *gr* knockout mutant, *gr* overexpression strain, and control strain (wild type carrying pJRD215) grown in S^0^ medium under zinc stress. *Acidithiobacillus caldus* (wild) represents the wild type, Δ*gr* represents the *gr* knockout mutant, *A. caldus* (pJRD215) represents the control strain, and *A. caldus* (pJRD215-*tac-gr*) represents the *gr* overexpression strain of *A. caldus* MTH-04, respectively. The concentrations of zinc are indicated as well. **(A)** 0 mM Zn^2+^; **(B)** 40 mM Zn^2+^; **(C)** 80 mM Zn^2+^; **(D)** 160 mM Zn^2+^. OD_600nm_ indicates the optical density at 600 nm, all measurements were performed in triplicate, and error bars correspond to the standard deviations.

The zinc tolerance of *A. caldus* strains was investigated as well. The increased growth inhibitions with the concentration of zinc ions were also observed in all *A. caldus* strains. Moreover, the Δ*gr* mutant grew lowest with a longer growth delay, while the *gr* overexpression strain grew highest with a shorter growth delay under the same concentration of zinc ions (Figure 8). The above results indicated the important role of the *gr* gene in zinc tolerance in *A. caldus* MTH-04.

### 3.3. Transcriptional analysis of *gr* knockout and overexpression strains

To further understand how *gr* may influence the expression patterns of glutathione-related genes in *A. caldus*, the relative mRNA levels of these genes were measured using the *gr* knockout strain with the wild type as the control or using the *gr* overexpression strain with the wild type carrying vacant pJRD215 plasmid as the control using the RT-qPCR measurement method. Without heavy metal stress, *gr* knockout resulted in increased expression levels of glutathione synthetase and glutathione peroxidase, while *gr* overexpression resulted in increased expression levels of glutathione reductase, thioredoxin reductase, and heterodisulfide reductase subunit C and decreased expression levels of peroxiredoxin, glutathione synthetase, and glutathione S-transferase (Table 4). These results suggest that the GR activity was involved in the glutathione system in *A. caldus* during elemental sulfur oxidization.

**Table 4:** Changes in the expression of genes in *Acidithiobacillus caldus* MTH-04 in *gr* knockout and *gr* overexpression strains.

**Locus**	**Gene description**	**Fold change (SD)** ^**a**^	
		Δ***gr***	**OE-** ***gr***
F0726_RS00165	Peroxiredoxin	1.1	1.0
F0726_RS02905	Peroxiredoxin, AhpC/Tsa family	1.4	**0.3**
F0726_RS04210	Glutathione reductase	–	**5.4**
F0726_RS04255	Thioredoxin reductase	1.3	**2.0**
F0726_RS04480	Glutathione synthetase	**3.4**	**0.4**
F0726_RS10185	Glutathione S-transferase	0.7	**0.4**
F0726_RS13530	Glutathione peroxidase	**2.2**	1.1
F0726_RS02000	Sulfur dioxygenase	1.7	1.2
F0726_RS12050	Heterodisulfide reductase subunit C	1.7	**3.1**

^a^Δ*gr* represents the *gr* knockout mutant; OE-gr represents the gr overexpression strain of *Acidithiobacillus caldus* MTH-04.

The expression of gene F0726_RS04210 was not detectable in gr knockout mutant and was shown as “–.” Fold change ≥ 2 and p-value ≤ 0.05 were considered to be upregulated, while fold change ≤ 0.5 and p-value ≤ 0.05 were considered to be downregulated and highlighted in bold.

When the strains were incubated with CuSO_4_, as shown in Table 5, most of the investigated genes were apparently upregulated in Δ*gr* compared with the wild type. On the other hand, the increased expression levels of the genes were somewhat different from others. For instance, the expression levels of peroxiredoxin, thioredoxin-disulfide reductase, and heterodisulfide reductase subunit C were increased after incubation for either 1 or 2 h, while the expression levels of thiol peroxidase and sulfur dioxygenase only increased after incubation for 1 h. When the *gr* overexpression strain was compared with the control strain (wild type carrying plasmid pJRD215), the expression levels of glutathione reductase and heterodisulfide reductase subunit C were increased after incubation for 1 and 2 h, respectively, while the expression levels of thioredoxin-disulfide reductase and sulfur dioxygenase were decreased after incubation for 1 h, and the expression levels of peroxiredoxin were decreased after incubation for 2 h, respectively.

**Table 5:** Changes in the expression of genes in *Acidithiobacillus caldus* MTH-04 in *gr* knockout and *gr* overexpression strains under copper stress.

**Locus**	**Gene description**	**Fold change (SD)** ^**a**^			
		Δ***gr***		**OE-** ***gr***	
		**Incubation for 1 h**	**Incubation for 2 h**	**Incubation for 1 h**	**Incubation for 2 h**
F0726_RS00165	Peroxiredoxin	**2.4**	1.4	0.7	0.6
F0726_RS02905	Peroxiredoxin, AhpC/Tsa family	**2.5**	**2.2**	0.8	**0.5**
F0726_RS04210	Glutathione reductase	–	–	**2.5**	1.6
F0726_RS04255	Thioredoxin reductase	**4.7**	**2.2**	**0.5**	0.8
F0726_RS10185	Glutathione S-transferase	0.6	0.8	0.8	1.3
F0726_RS13530	Glutathione peroxidase	1.7	**2.0**	1.5	1.1
F0726_RS02000	Sulfur dioxygenase	**2.9**	0.9	**0.5**	1.6
F0726_RS12050	Heterodisulfide reductase subunit C	**2.3**	**2.4**	1.4	**2.7**

^a^Δ*gr* represents the *gr* knockout mutant; OE-gr represents the gr overexpression strain of *Acidithiobacillus caldus* MTH-04.

The expression of gene F0726_RS04210 was not detectable in gr knockout mutant and was shown as “–.” Fold change ≥ 2 and p-value ≤ 0.05 were considered to be upregulated, while fold change ≤ 0.5 and p-value ≤ 0.05 were considered to be downregulated and highlighted in bold.

Table 6 presents the responses of the selected genes to ZnSO_4_. It shows that when compared with the wild type, the deletion of *gr* resulted in the upregulation of peroxiredoxin and heterodisulfide reductase subunit C and the downregulation of thioredoxin reductase, glutathione S-transferase, and sulfur dioxygenase after incubation for 1 h. The absence of *gr* also resulted in the upregulation of thiol peroxidase, peroxiredoxin, and thioredoxin reductase after incubation for 2 h. When compared with the control strain (wild type carrying plasmid pJRD215), the overexpression of *gr* resulted in the upregulation of glutathione reductase and heterodisulfide reductase subunit C after incubation for either 1 or 2 h and downregulation of peroxiredoxin after incubation for 2 h. The above results suggest that the GR activity was involved in heavy metal tolerance in *A. caldus*.

**Table 6:** Changes in the expression of genes in *Acidithiobacillus caldus* MTH-04 in *gr* knockout and *gr* overexpression strains under zinc stress.

**Locus**	**Gene description**	**Fold change (SD)** ^**a**^			
		Δ***gr***		**OE-** ***gr***	
		**Incubation for 1 h**	**Incubation for 2 h**	**Incubation for 1 h**	**Incubation for 2 h**
F0726_RS00165	Peroxiredoxin	0.9	**2.5**	0.6	1.0
F0726_RS02905	Peroxiredoxin, AhpC/Tsa family	**6.1**	**2.2**	0.6	**0.5**
F0726_RS04210	Glutathione reductase	–	–	**5.3**	**3.0**
F0726_RS04255	Thioredoxin reductase	**0.5**	**2.1**	1.3	1.0
F0726_RS10185	Glutathione S-transferase	0.1	1.1	1.2	1.0
F0726_RS13530	Glutathione peroxidase	1.3	1.6	0.9	1.0
F0726_RS02000	Sulfur dioxygenase	**0.3**	1.8	1.6	1.7
F0726_RS12050	Heterodisulfide reductase subunit C	**3.7**	1.7	**6.3**	**6.2**

^a^Δ*gr* represents the *gr* knockout mutant; OE-gr represents the gr overexpression strain of *Acidithiobacillus caldus* MTH-04.

The expression of gene F0726_RS04210 was not detectable in gr knockout mutant and was shown as “–.” Fold change ≥ 2 and p-value ≤ 0.05 were considered to be upregulated, while fold change ≤ 0.5 and p-value ≤ 0.05 were considered to be downregulated and highlighted in bold.

## 4. Discussion

A putative *gr* gene (F0726_RS04210) was detected in the genome of *A. caldus* MTH-04 by conducting a BLASTP search. The proteins with GR activity contain conserved functional motifs, including an NADPH binding site, a GSSG binding site, and a redox-active disulfide bond domain (Figure 3). Most of the GR homologs contain a highly conserved NADPH binding site sequence (RX5R). Moreover, the first arginine residue in the RX5R motif is conserved up to 100%, while the last arginine residue is replaced by different residues in different strains. In F0726_RS04210, a histidine residue replaces the last conserved arginine residue (RX5H). Similar phenomena were also reported in *F. noatunensis* subsp. *orientalis* str. Toba 04 (RX5M), *S. meliloti* (RX5S), and *Nostoc* sp. PCC7120 (RX5K). Replacing arginine with other basic amino acids (H or K) may affect the affinity of NADPH and result in a wider range of electronic sources, such as NADPH and NADH. In addition, the evolutionary relationship of F0726_RS04210 is close to the glutathione reductase from *E. coli* K12. Finally, we detected GR activity in the protein of F0726_RS04210. The above results indicate that F0726_RS04210 is a GR and may play a similar role as the reported GRs.

It is well established that *A. caldus* can obtain energy by oxidizing reduced inorganic sulfur compounds (RISCs) during chemolithoautotrophic growth in acidic environments. Oxidation of RISCs requires elemental sulfur (S^0^) as the initial primary and intermediate metabolite; however, limited information is available on the activation of elemental sulfur. Previous studies have proposed that elemental sulfur may be activated by GSH in *Acidithiobacillus* (Silver and Lundgren, 1968). However, the Δ*gr* mutant of *A. caldus* still grew well when using elemental sulfur as the sole energy substrate. The results indicate that GR is not fatal in the oxidation of elemental sulfur in *A. caldus*.

In biometallurgy, ore leaching microorganisms are in an environment of high osmotic pressure and high concentration of heavy metals. Our recent research has explained the essential role of OmpR in *A. caldus* adapting to the high osmolarity (Chen et al., 2022), but little is known about the mechanism of heavy metal resistance. A copper-sensitive operon repressor was identified in *A. caldus*, which might be involved in putative copper resistance mechanisms (Hou et al., 2021). Due to the lack of effective genetic tools, this specific mechanism still needs to be verified. Heavy metals accumulate during the bioleaching process, and the stress tolerance process will result in reactive oxygen species (ROS) (Stadtman and Oliver, 1991; Natarajan et al., 1994). Recently, GR was reported to participate in the heavy metal tolerance of *A. ferrooxidans* (Xia et al., 2011; Zheng et al., 2015, 2016), so the role of GR in the heavy metal tolerance of *A. caldus* was investigated in this research. Deletion of *gr* resulted in increased sensitivity to heavy metals, while the overexpression of *gr* enhanced tolerance to heavy metals, which suggests the involvement of the *gr* gene in heavy metal tolerance in *A. caldus* MTH-04. Moreover, enzymes involved in the antioxidant pathway (for instance, thioredoxin reductase) and GSH-producing pathway (for instance, glutathione synthetase and heterodisulfide reductase subunit C) were altered when *gr* was deleted or overexpressed in *A. caldus* under heavy metal stress. Previous studies also reported that GR plays a key role in heavy metal tolerance by keeping high GSH/GSSG ratios (Schirmer et al., 1989; Creissen et al., 1994; Mullineaux and Creissen, 1997). The results indicate that GR may play a key role in heavy metal tolerance in *A. caldus* by sustaining the reduced status of glutathione.

## 5. Conclusion

We detect a *gr* gene in *A. caldus* and provide the report characterizing the *gr* gene by constructing a *gr* knockout mutant and a *gr* overexpression strain. We found that *gr* knockout results in increased sensitivity to heavy metals (Cu^2+^ and Zn^2+^) and revealed the strong correlations between GR and the antioxidant pathway in *A. caldus*. Finally, we propose the function of GR is to play an important role in heavy metal tolerance. Our findings provide a template for further investigation of GR in other microorganisms and can be further used to construct improved bioleaching strains.

## Data availability statement

The original contributions presented in the study are included in the article/supplementary material, further inquiries can be directed to the corresponding authors.

## Author contributions

YS, YY, and XiaoL: methodology. JianqiL: formal analysis. XianL: validation. JianquL and XP: investigation and supervision. WW: initial draft and revised draft writing and editing. JianquL and XP: funding acquisition. All authors have read and agreed to the published version of the manuscript.
